# Integrated analysis of N6-methyladenosine- and 5-methylcytosine-related long non-coding RNAs for predicting prognosis in cervical cancer

**DOI:** 10.1186/s41065-024-00336-w

**Published:** 2024-09-16

**Authors:** Jie Gao, Xiuling Zhang, Anqi Xu, Wei Li, HaiYan Gao

**Affiliations:** 1 Department of Laboratory Medicine, The Sixth Affiliated Hospital of Harbin Medical University, 998 Aiying Street, Songbei District, Harbin, 150028 Heilongjiang Province China; 2 Department of Rheumatism and Immunology, The 2nd Affiliated Hospital of Harbin Medical University, 246 Xuefu Road, Nangang District, Harbin, 150001 Heilongjiang Province China

**Keywords:** N6-methyladenosine, 5-methylcytosine, Long non-coding RNAs, Tumor microenvironment, Risk score, Nomogram

## Abstract

**Background:**

N6-methyladenosine (m^6^A) and 5-methylcytosine (m^5^C) play a role in modifying long non-coding RNAs (lncRNAs) implicated in tumorigenesis and progression. This study was performed to evaluate prognostic value of m^6^A- and m^5^C-related lncRNAs and develop an efficient model for prognosis prediction in cervical cancer (CC).

**Methods:**

Using gene expression data of TCGA set, we identified m^6^A- and m^5^C-related lncRNAs. Consensus Clustering Analysis was performed for samples subtyping based on survival-related lncRNAs, followed by analyzing tumor infiltrating immune cells (TIICs). Optimal signature lncRNAs were obtained using lasso Cox regression analysis for constructing a prognostic model and a nomogram to predict prognosis.

**Results:**

We built a co-expression network of 23 m^6^A-related genes, 15 m^5^C-related genes, and 62 lncRNAs. Based on 9 m^6^A- and m^5^C-related lncRNAs significantly associated with overall survival (OS) time, two molecular subtypes were obtained, which had significantly different OS time and fractions of TIICs. A prognostic model based on six m^6^A- and m^5^C-related signature lncRNAs was constructed, which could dichotomize patients into two risk subgroups with significantly different OS time. Prognostic power of the model was successfully validated in an independent dataset. We subsequently constructed a nomogram which could accurately predict survival probabilities. Drug sensitivity analysis found preferred chemotherapeutic agents for high and low-risk patients, respectively.

**Conclusion:**

Our study reveals that m^6^A- and m^5^C-related lncRNAs are associated with prognosis and immune microenvironment of CC. The m^6^A- and m^5^C-related six-lncRNA signature may be a useful tool for survival stratification in CC and open new avenues for individualized therapies.

**Supplementary Information:**

The online version contains supplementary material available at 10.1186/s41065-024-00336-w.

## Background

Cervical cancer (CC) is a highly heterogeneous cancer and ranks fourth in incidence and mortality in females worldwide, with cervical squamous cell carcinoma (CESC) as the most common type [1]. CC incidence has experienced an evident increase in recent years because of the availability of effective screening programs [2]. Human papilloma virus (HPV) is a well-documented etiological factor of CC, and thus receiving HPV vaccine remains a primary method for CC prevention [3]. CC patients are commonly treated with combinations of surgical resection, chemotherapy, radiotherapy or other therapies [4]. Nonetheless, the long-term survival and prognosis of advanced CC remains unsatisfactory [5]. Therefore, there is an evident interest in gaining a deeper understanding of molecular mechanisms behind CC carcinogenesis and discovering promising prognostic signatures capable of predicting clinical outcomes of patients reliably and accurately.

Long non-coding RNAs (LncRNAs), a group of non-coding RNAs longer than 200 nucleotides, participate in a variety of biological processes involved in tumorigenesis and progression [6, 7]. A significant body of literature has supported important biological roles of lncRNAs in CC progression, invasion and metastasis, and their potentials as prognostic biomarkers [8, 9]. N6-methyladenosine (m^6^A) and 5-methylcytosine (m^5^C) are two important forms of modifications to messenger RNA (mRNAs) and non-coding RNAs (ncRNAs), playing a role in diverse functions, such as RNA splicing and translation. Either m^6^A or m^5^C modification has three classes of components: intracellular methyltransferases (“writers”), demethylases (“erasers”), and signal transducers (“readers”) [10]. m^6^A methyltransferase METTL3 up-regulation has been observed in CC, and may serve as prognostic biomarkers [11]. m^6^A reader YTHDF1 plays a oncogenic role and is related to poor prognosis of CC patients [12]. Moreover, growing evidences are highlighting m^6^A-related genes and m^6^A-related lncRNAs as potential prognostic biomarkers in CC [13, 14]. Substantial evidences have revealed that m^5^C modification is implicated in tumorigenesis, cancer migration, and metastasis of a large number of cancers including CC [15–17]. However, there is a lack of studies on characterization of biological roles of m^5^C modification and m^5^C-related lncRNAs in the pathophysiology of CC.

Biological significance of m^6^A and m^5^C modifications implies the potential of m^6^A-and m^5^C-related lncRNAs to be used for prognostic purposes in CC. To decipher the regulatory networks of m^6^A-and m^5^C-related lncRNAs in tumorigenesis and progression of CC and explore their prognostic value, we identified m^6^A-and m^5^C-related lncRNAs in CESC samples downloaded from The Cancer Genome Atlas (TCGA). Out of them, we determined survival-related lncRNAs and subsequently, revealed two molecular subtypes. Furthermore, with prognostic lncRNAs we constructed a risk score model and a nomogram to predict prognosis in CESC. In addition, we unraveled associations of m^6^A-and m^5^C-related lncRNAs with tumor infiltrating immune cells (TIICs) in tumor microenvironment (TME) as well.

## Methods

### Data sources

Gene expression data (log2 (FPKM + 1), Illumina HiSeq 2000 RNA Sequencing platform) of 291 CC samples and corresponding clinicopathological characteristics were downloaded from TCGA database (https://portal.gdc.cancer.gov/) and were used as the training set. The validated set of this study used GSE44001 dataset [18] downloaded from gene expression omnibus (GEO) database (https://www.ncbi.nlm.nih.gov/geo/), including gene expression data and survival information of 300 CC samples (Illumina HumanHT-12 WG-DASL V4.0 R2 expression beadchip).

### Identification of m6A- and m5C-related lncRNAs

According to a recently published study [19], 23 m^6^A-related genes and 15 m^5^C-related genes were identified (Supplemental Table 1). Expression levels of genes associated with m^5^C and m^6^A were extracted from the TCGA dataset. Using the cor function in R3.6.1 (http://77.66.12.57/R-help/cor.test.html), Pearson correlation coefficients (PCC) were calculated between the expression levels of 23 m6A-related genes, 15 m5C-related genes, and all annotated lncRNAs. The m6A- and m5C-related lncRNAs were identified using a threshold of |PCC| > 0.3 and a significance p-value < 0.05. Finally, we identified lncRNAs significantly associated with both m5C and m6A genes and selected those that were significantly correlated with both. A co-expression network between m5C and m6A genes and their commonly associated lncRNAs was constructed and visualized using Cytoscape 3.6.1 [20] (https://cytoscape.org/).

### Consensus clustering analysis

Based on the expression levels of m6A- and m5C-related lncRNAs in CESC tumor samples from the TCGA training dataset, univariate Cox regression analysis was performed using the survival package Version 2.41-1 (http://bioconductor.org/packages/survivalr/) in R 3.6.1 to identify lncRNAs significantly associated with survival prognosis, with a significance threshold set at *P* < 0.05. Based on expression levels of these survival-related lncRNAs, CC samples in the TCGA set were separated into different subtypes by performing Consensus Clustering Analysis using ConsensusClusterPlus package [21] (version 1.54.0, http://www.bioconductor.org/packages/release/bioc/html/ConsensusCluster Plus.html) in R language. The parameters were set as d, maxK = 10, reps = 50, pItem = 0.8, pFeature = 1, clusterAlg="hc”, distance="pearson”. Kaplan- Meier (KM) survival curves were plotted for different subtypes using *survival* package.

### Analysis of immune infiltration

R3.6.1 GSVA (http://www.bioconductor.org/packages/release/bioc/html/GSVA.html) Version Version 1.36.3, which was based on single sample gene set enrichment analysis (ssGSEA) algorithm was adopted to evaluate the proportion of TIICs in TCGA samples. Subsequently, we performed differential distribution comparisons of TIIC proportions across different subtypes using group t-tests in R3.6.1, with a significance threshold set at a p-value of less than 0.05. Finally, we analyzed the correlation between the expression levels of prognostically significant lncRNAs and the TIIC types that showed significant distribution differences.

### Establishment of m6A- or m5C-related lncRNA signature for survival prediction

Briefly, out of the survival-related lncRNAs, the optimal prognostic lncRNAs were selected by performing Lasso Cox regression analysis [22] using *lars* package (https://cran.r-project. org/web/packages/lars/index.html) in R. The optimal lambda value was determined by performing a 10-fold cross-validation. Based on LASSO coefficients and expression levels of the optimal signature lncRNAs, prognostic model was constructed using the following formula:

Risk score (RS) = ∑Coef_lncRNAs_×Exp_lncRNAs_.

Where Coef_lncRNAs_ represents the estimated LASSO coefficient of lncRNAs; Exp_lncRNAs_ represents expression level of lncRNAs.

With median risk score as the cutoff, samples were divided into two risk subgroups in the TCGA set. KM survival curves were plotted for different risk subgroups and compared using log-rank tests. Accuracy, sensitivity and specificity of the prognostic model were assessed using receiver operating characteristic (ROC) curve. Predictive performance of the prognostic score model was validated using GSE44001 dataset.

### Identification of independent prognostic factors and nomogram construction

Clinical factors of patients in the TCGA set were subjected to uni-variable and multi-variable Cox regression analysis to identify independent prognostic factors using *survival* package in R (log-rank p-value < 0.05). Nomogram [23] that could provide a predicted probability was generated using *rms* package (version 5.1-2) in R (https://cran.r-project.org/web/packages/rms/index.html).

### Drug sensitivity analysis

Based on gene expression profiles of CC samples in the TCGA set and chemotherapeutic agents downloaded from Genomics of Drug Sensitivity in Cancer (GDSC) [24] database (https://www.cancerrxgene.org/), sensitivity of CESC patients in high-risk and low-risk groups to chemotherapeutic drugs was assessed by predicting half-maximal inhibitory concentration (IC50) using *pRRophetic* [25] package in R (http://127.0.0.1:22402/doc/html/Search?objects=1&port=22402). IC50 values were compared between different risk groups using Students’ t test to assess differential therapeutics effects of chemotherapeutic drugs.

### Function annotation analyses

Differentially expressed genes (DEGs) were screened between different risk groups of the TCGA set using *limma* [26] package in R (https://bioconductor.org/packages/release/bioc/html/limma.html) with FDR < 0.05 and |log_2_FC|>0.5 as the significance cutoff. Gene ontology (GO) function and Kyoto Encyclopedia of Genes and Genomes (KEGG) pathway enrichment analyses were conducted using The Database for Annotation, Visualization and Integrated Discovery (DAVID) tool (https://david.ncifcrf.gov/, version 6.8) with the strict cutoff at FDR < 0.05.

## Results

### A co-expression network was constructed including 23 m^6^A genes, 15 m^5^C genes and 343 lncRNAs

**Fig. 1:**
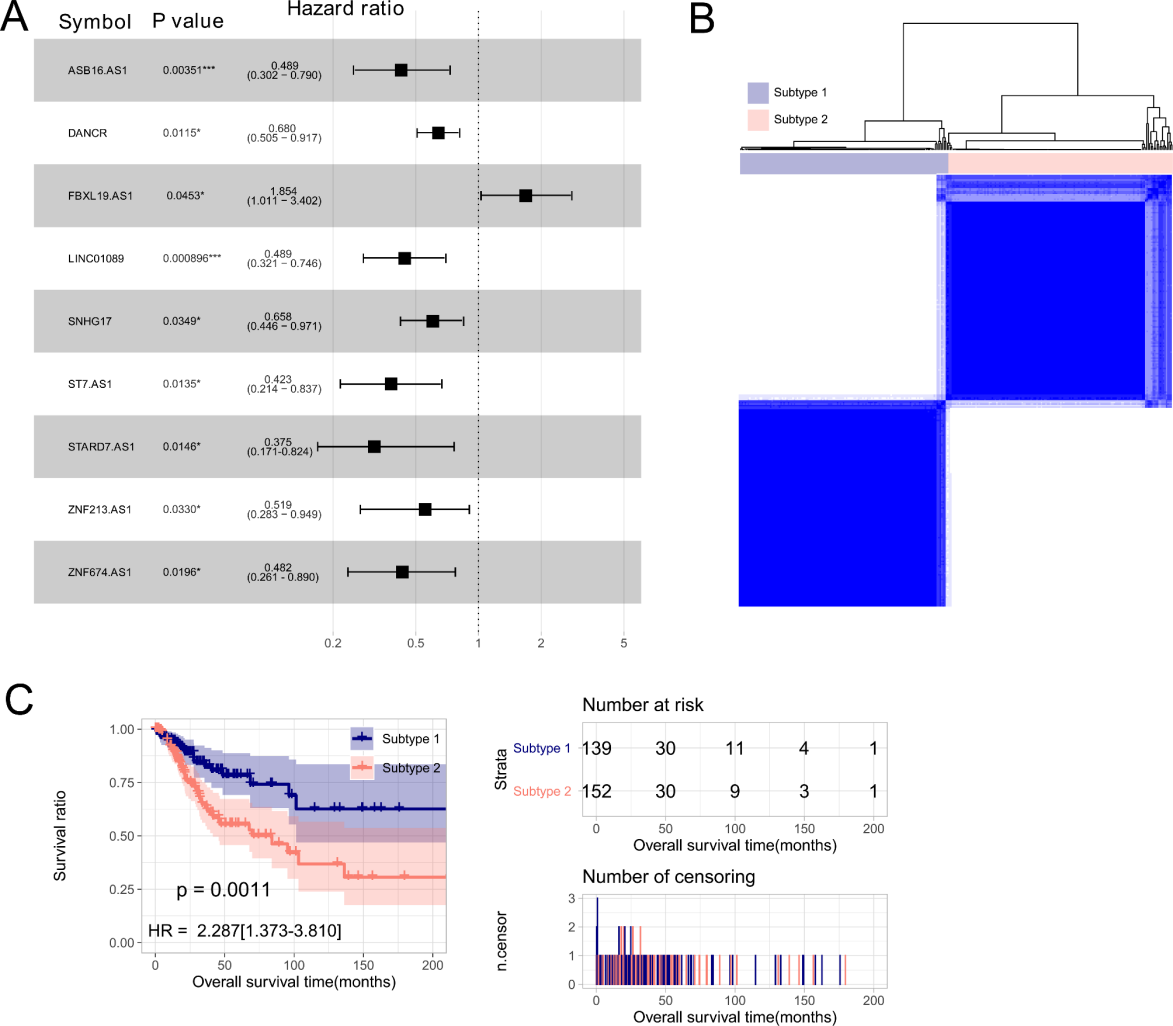
Identification of two subtypes with signficantly different survival time. (**A**) Forest plot of 9 survival-related lncRNAs in uni-variable Cox regression analysis; (**B**) samples are classified into two subtypes (subtype 1 and 2) by Consensus Clustering Analysis; (**C**) Kaplan- Meier curvers of two subtypes

As mentioned in Method section, we recognized 23 m^6^A-related genes and 15 m^5^C-related genes, and extracted 343 common lncRNAs from both the TCGA set and GSE44001 dataset. By performing Pearson correlation analysis, we obtained 202 m^6^A genes-lncRNAs pairs and 264 m^5^C genes-lncRNAs pairs, which shared 62 common lncRNAs. With these m^6^A genes, m^5^C genes and 62 m^6^A and m^5^C-related lncRNAs, a co-expression network was constructed (Figure S1). Out of the 62 m^6^A and m^5^C-related lncRNAs, 9 lncRNAs were significantly related to OS time in uni-variate Cox regression analysis (Fig. 1A).

### Two molecular subtypes were obtained based on nine survival-related lncRNAs

Based on expression levels of the 9 survival-related lncRNAs in the TCGA set, samples were categorized into subtype 1 (*N* = 139) and subtype 2 (*N* = 152) through performing Consensus Clustering Analysis (Fig. 1B). KM survival curves showed that subtype 1 showed significantly longer OS time than subtype 2 (*p* = 0.0011, HR = 2.287 (1.373–3.810), Fig. 1C).

### Characterization of tumor infiltrating immune cells in different subtypes

**Fig. 2:**
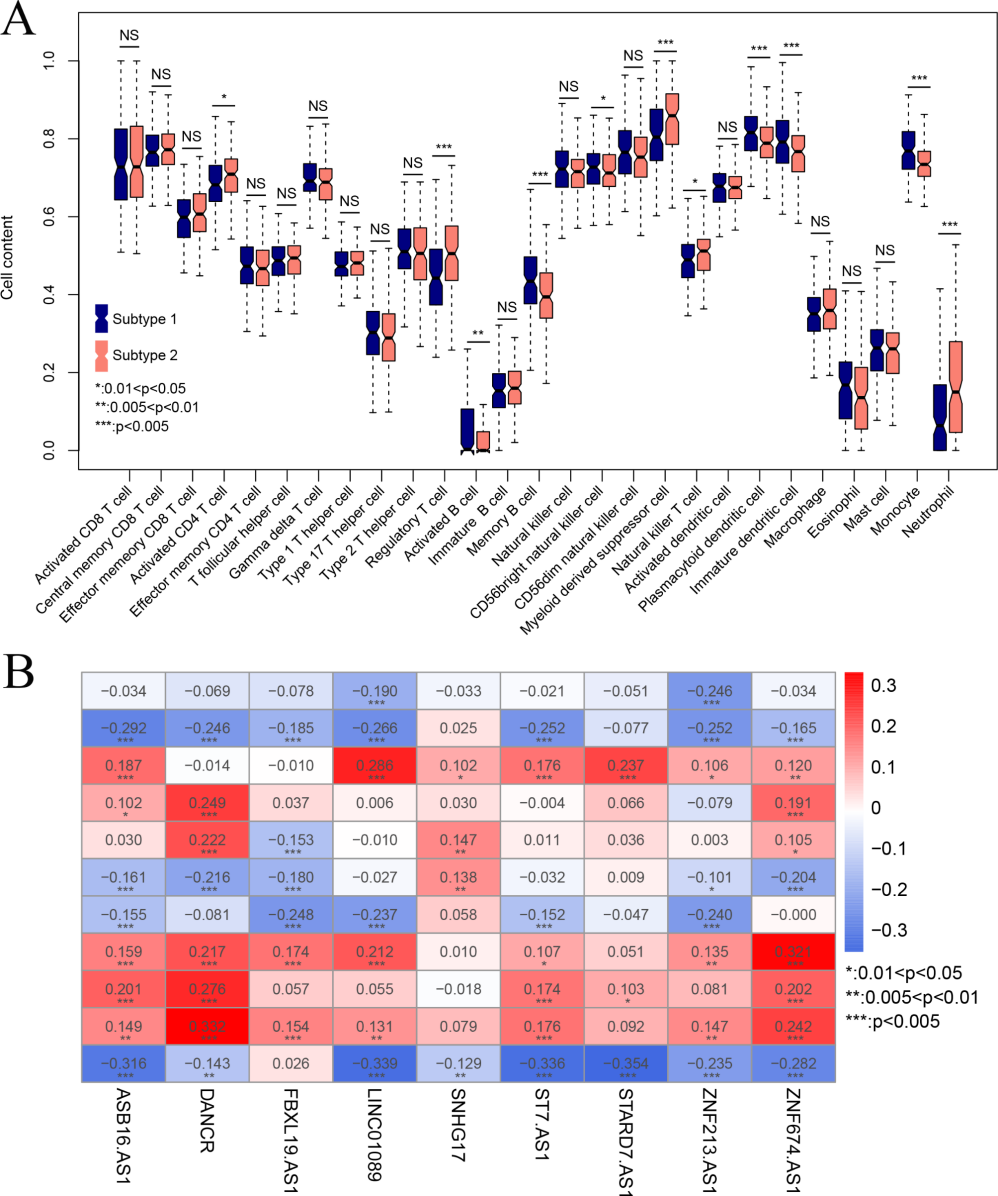
Characterization of tumor infiltrating immune cells in different subtypes. (**A**) Comparative analysis of fractions of tumor infiltrating immune cells between two subtypes; (**B**) Pearson correlation coefficients of 9 survival-related lncRNAs with 11 infiltrating immune cells

In order to investigate the characteristics of TME and TIICs of the two molecular subtypes, we analyzed and compared fractions of different TIICs between them. As shown in Fig. 2A, subtype 1 had significantly lower levels of activated CD4 + T cells, regulatory T cells, myeloid derived suppressor cells, memory B cells, natural killer T cells and neutrophils, and higher levels of activated B cells, plasmacytoid dendritic cells, CD56 + bright natural killer cells, immature dendritic cells, and monocytes (p-value < 0.05). Moreover, PCCs between the 9 survival-related lncRNAs and 11 immune cells were shown in a heatmap (Fig. 2B). Fractions of activated CD4 + T cells, and neutrophils were negatively correlated with expression levels of all nine lncRNAs. On the contrary, fractions of plasmacytoid dendritic cells, and monocytes were positively correlated with expression levels of all nine lncRNAs.

### A prognostic model based on six m^6^A and m^5^C-related lncRNAs was constructed and successfully validated in an independent set

**Fig. 3:**
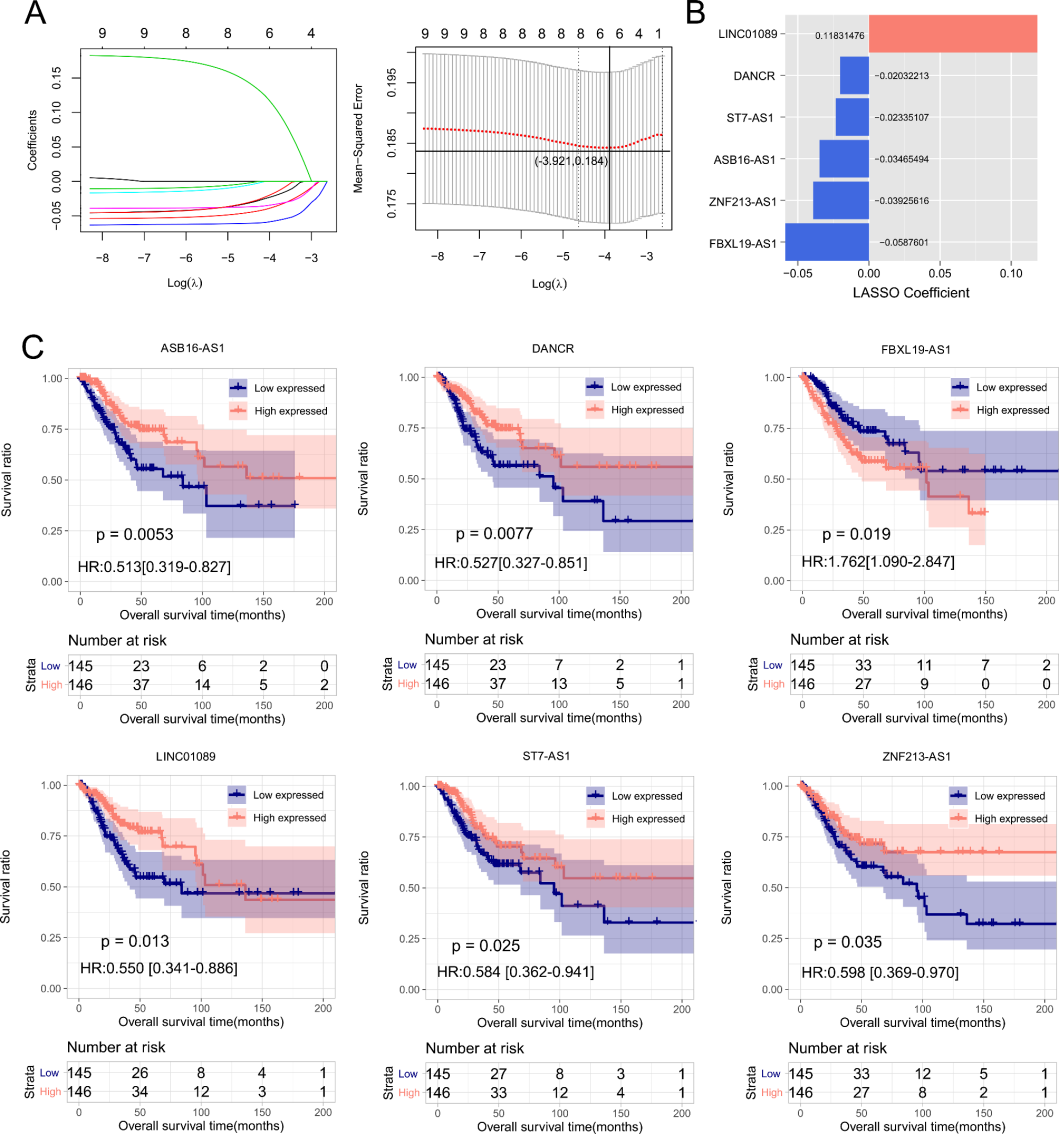
LASSO Cox regression analysis. (**A**) Performance indicators of LASSO regression analysis (**B**) LASSO coefficients of 6 signature lncRNAs. (**C**) Kaplan-Meier curves for patients stratified by each signature lncRNA. Patients are divided into high-expression group and low-expression groups according to median expression level of a signature lncRNA (ASB16-AS1, DANCR, LINC01089, ST7-AS1, FBXL19-AS1 or ZNF213-AS1)

LASSO Cox regression was performed based on the aforementioned nine survival-related lncRNAs. As a result, six optimal lncRNAs were obtained, consisting of ASB16-AS1, DANCR, FBXL19-AS1, LINC01089, ST7-AS1, and ZNF213-AS1 (Fig. 3A-B). Based on LASSO coefficients and expression levels of the six optimal lncRNAs in the TCGA set, risk score was calculated for each sample using the following formula:

RS = (-0.0587601)*Exp_LINC01089_ + (-0.03925616)*Exp _DANCR_ +(-0.03465494)*Exp _ST7−AS1_+ (-0.02335107)*Exp _ASB16−AS1_ +(-0.02032213)*Exp _ZNF213−AS1_+(0.11831476)*Exp _FBXL19−AS1_.

**Fig. 4:**
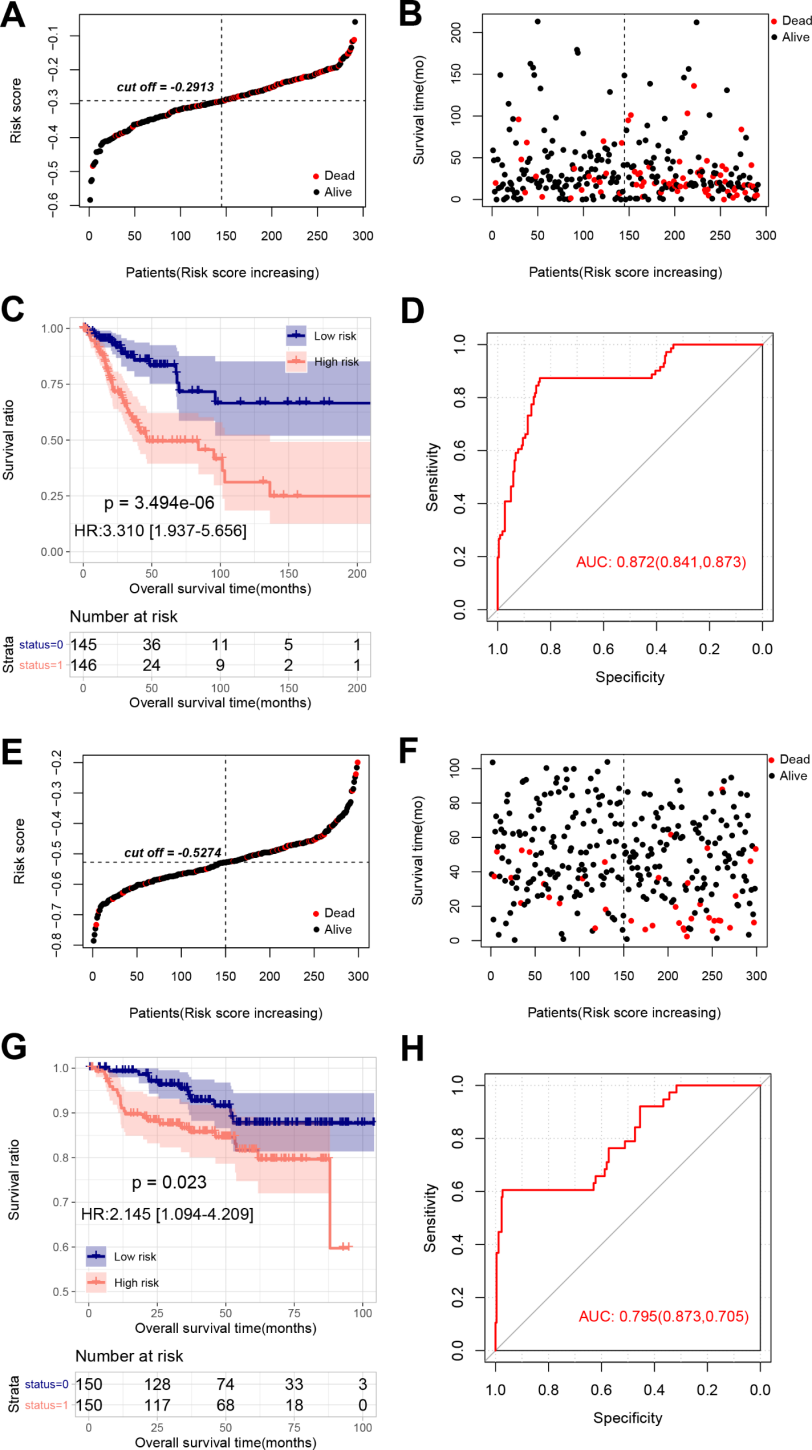
Performance of the risk model in TCGA set and validation set. Risk score distribution (**A**), relationship of risk score and survival time (**B**), KM curves of high-risk and low risk patients (**C**) and ROC curve (D) of patients in high-risk and low-risk subgroups in the TCGA set. Risk score distribution (**E**), relationship of risk score and survival time (**F**), KM curves of high-risk and low risk patients (**G**) and ROC curve (**H**) of patients in high-risk and low-risk subgroups in the validation set (GSE44001)

With regard to ASB16-AS1 (*p* = 0.0053), DANCR (*p* = 0.0077), LINC01089 (*p* = 0.013), ST7-AS1 (*p* = 0.025), or ZNF213-AS1 (*p* = 0.035), high-expressed patients had significantly longer OS time compared to low-expressed patients (Fig. 3C). Conversely, patients with low-expressed FBXL19-AS1 had longer OS time in comparison with patients with high-expressed FBXL19-AS1 (*p* = 0.019, Fig. 3C). In the TCGA set, with median risk score (-0.2913) as cutoff, tumor samples were separated into a high-risk group and a low-risk group (Fig. 4A-B). As depicted in Fig. 4C-D, OS time was significantly longer in low-risk patients compared to high-risk patients (p-value = 3.494e-06, HR = 3.310 (1.937–5.565)), with an AUC of 0.872 (0.841, 0.873). Similarly, the risk score model was applied to GSE44001 dataset. As shown in Fig. 4E-H, patients were dichotomized into high-risk and low-risk patients with significantly different OS time (p-value = 0.023, HR = 2.145 (1.094–4.209)) and an AUC of 0.795 (0.873, 0.705).

**Fig. 5:**
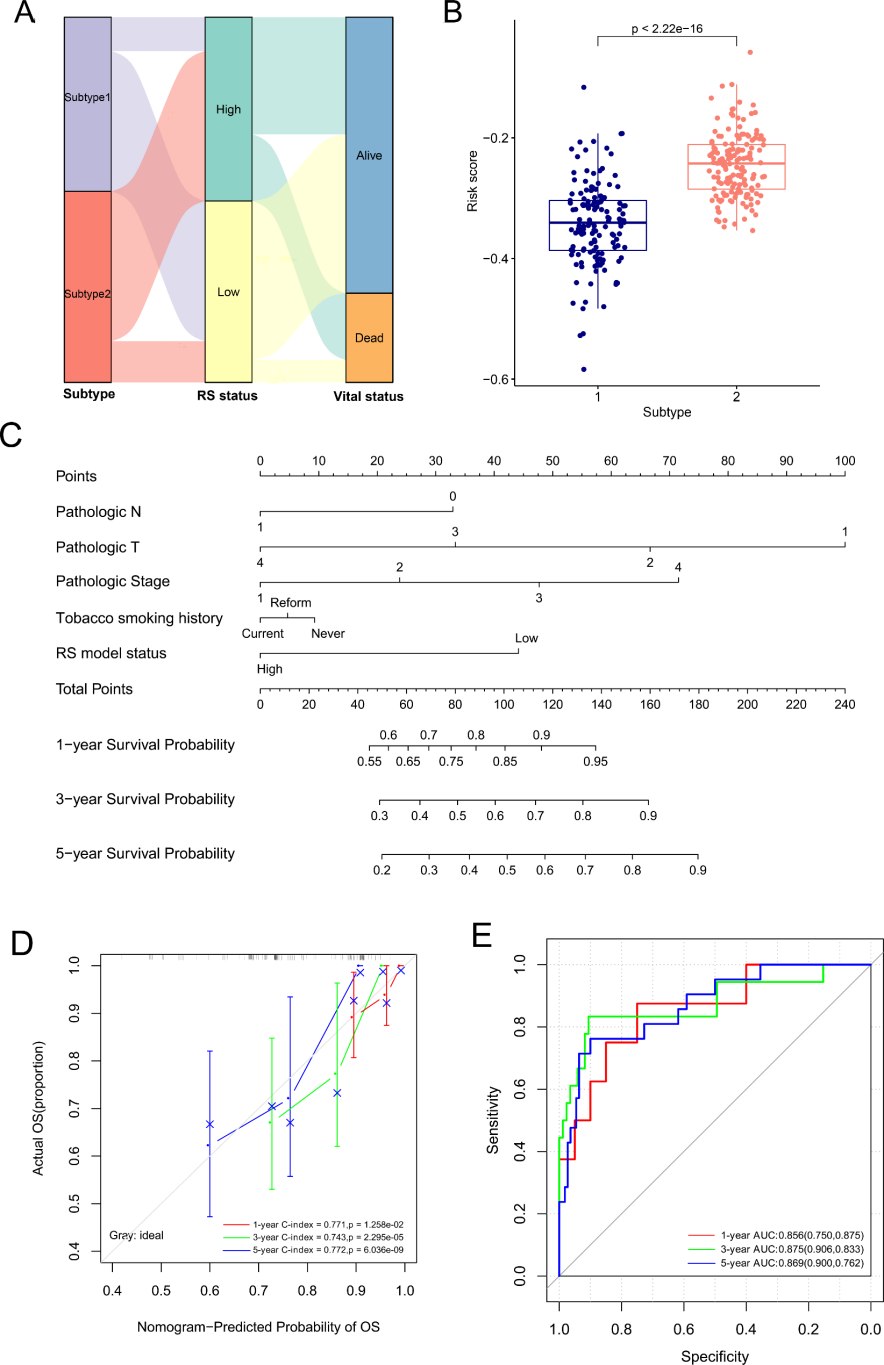
Associations of molecular subtypes with risk score and nomogram construction. (**A**) Sankey diagram of molecular subtypes, risk score status and vital status of patients; (**B**) The risk scores of two subtypes were significantly different; (**C**) a nomogram for predicting 1-, 3- and 5- year survival probability in the TCGA set; (**D**) Calibration plots of the nomogram; (**E**) ROC curves for predicting 1-, 3- and 5- year survival probability

Sankey diagram was used to analyze the relationships of the above-mentioned two subtypes, two risk subgroups and vital status of CC patients in the TCGA set. The majority of subtype 1 samples were classified into the low-risk group and had better prognosis, while the majority of subtype 2 samples were classified into the high-risk group and had poor prognosis (Fig. 5A). Risk scores were significantly decreased in subtype 1 samples compared to subtype 2 samples (Fig. 5B, p-value < 2.22e-16). These results reveal that the subtyping results based on the 9 survival-related lncRNAs were consistent with the risk stratification results based on the six-m^6^A and m^5^C-related lncRNAs signature.

### A nomogram was constructed integrating risk score with prognostic factors

Uni-variable and multi-variable Cox regression analysis was carried out for clinical factors of patients in the TCGA set. As shown in Table 1, Pathologic N (p-value = 2.19E-03, 95%CI = 1.409–5.596), Pathologic T (p-value = 2.31E-05, 95%CI = 1.371–2.452), Pathologic Stage (p-value = 2.86E-04, 95%CI = 1.195–1.852), Tobacco smoking history (p-value = 6.30E-03, 95%CI = 1.081–1.904) and RS model (p-value = 3.49E-06, 95%CI = 1.937–5.656) were significantly associated with OS time of CC patients. Furthermore, RS model was an independent prognostic factor (p-value = 4.34E-02, 95%CI = 1.027–5.861).

**Table 1:** Uni-variable and multi-variable Cox regression analysis of clinical factors

Clinical characteristics	Uni-variables cox			Multi-variables cox		
	HR	95%CI	*P*-value	HR	95%CI	*P*
Age(years, mean ± SD)	1.017	0.999–1.035	5.68E-02	-	-	-
Pathologic_M (M0/M1)	3.671	1.229–10.96	2.56E-01	-	-	-
Pathologic_N (N0/N1)	2.808	1.409–5.596	2.19E-03	2.222	0.969–5.097	5.95E-02
Pathologic_T (T1/T2/T3/T4)	1.833	1.371–2.452	2.31E-05	1.852	0.890–3.853	9.91E-02
Pathologic_stage (I/II/III/IV)	1.488	1.195–1.852	2.86E-04	0.608	0.305–1.212	1.58E-01
Neoplasm histologic grade (G1/G2/G3)	0.976	0.643–1.535	9.76E-01	-	-	-
Tobacco smoking history (Never/Reform/Current)	1.435	1.081–1.904	6.30E-03	1.172	0.740–1.856	4.98E-01
Prognostic model (High/Low)	3.31	1.937–5.656	3.49E-06	2.454	1.027–5.861	4.34E-02

SD, standard deviation; HR, hazard ratio; CI, confidence interval

Combining risk score model with pathologic N, pathologic T, pathologic Stage, and tobacco smoking history, a nomogram was built to predict survival probabilities in CC patients (Fig. 5C). Calibration curves showed that 1-year C-index was 0.771, with a p-value of 1.258e-02; 3-year C-index = 0.743, with a p-value of 2.295e-05; 5-year C-index = 0.772, with a p-value of 6.036e-09 (Fig. 5D). Moreover, in ROC curves, 1-year, 3-year and 5-year AUC was 0.856 (0.750, 0.875), 0.875 (0.906, 0.833), 0.869 (0.900, 0.762), separately (Fig. 5E). These results collectively suggest that the nomogram model has high accuracy in predicting 1-, 3-, 5-year survival in CC patients.

### Differential sensitivity of high-risk and low-risk patients to chemotherapeutic agents

**Fig. 6:**
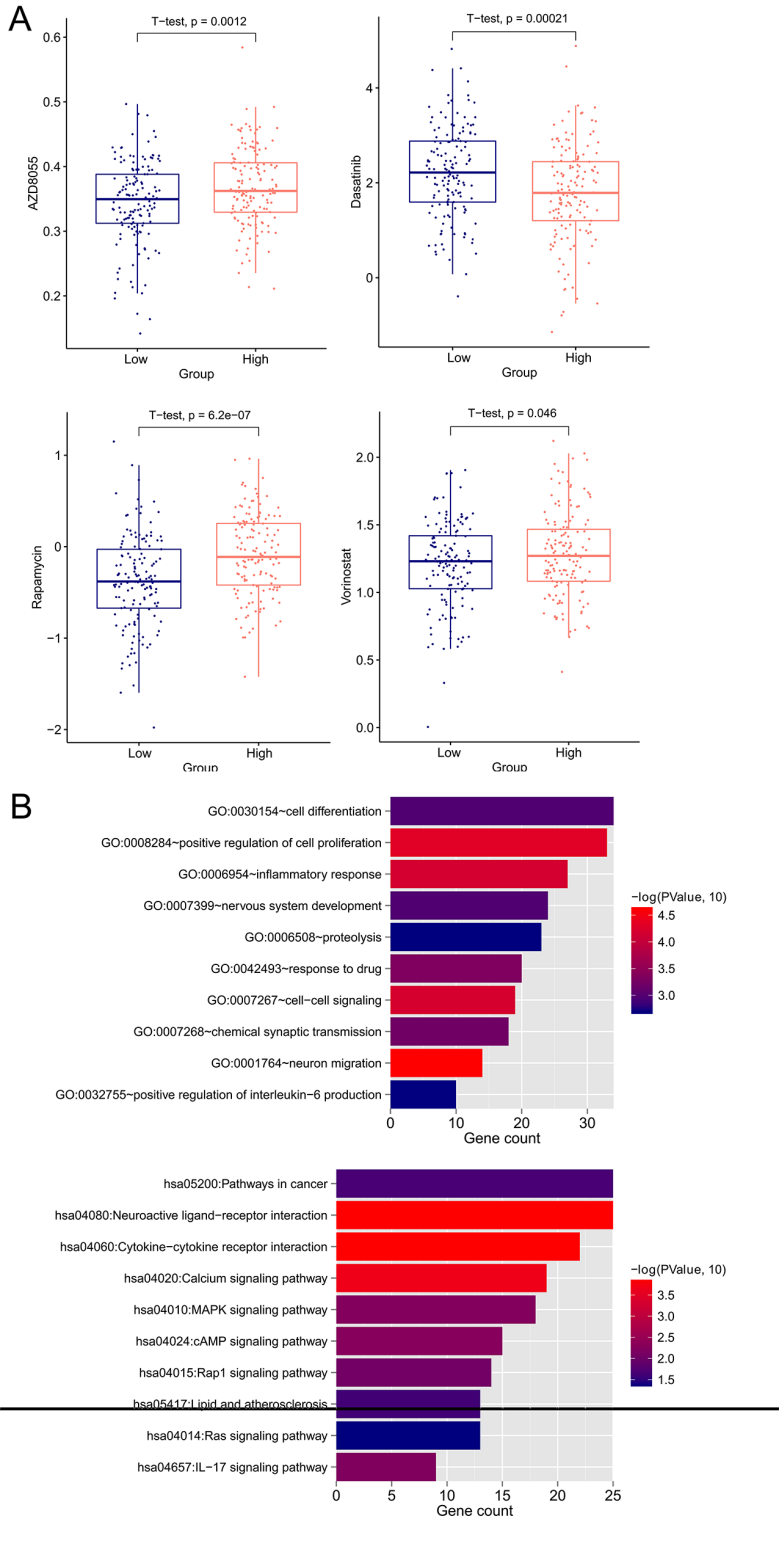
Drug sensitivity and functional enrichment analysis. (**A**) IC50 values of AZD8055, Rapamycin, Vorinostat and Dasatinib between high-risk and low-risk patients in the TCGA set. (**B**) Top 10 GO biological processes and top 10 KEGG signaling pathways

We compared sensitivities of high-risk and low-risk patients to 21 chemotherapeutic agents (Axitinib, AZD6482, AZD7762, AZD8055, Bortezomib, Camptothecin, Cisplatin, Cytarabine, Dasatinib, Erlotinib, Gefitinib, Gemcitabine, GSK269962A, Lapatinib, Nilotinib, Paclitaxel, Rapamycin, Sorafenib, Vinblastine, Vinorelbine, and Vorinostat) in the TCGA set. Low-risk patients were more sensitive to AZD8055 (p-value = 0.0012), Rapamycin (p-value = 6.2e-07) and Vorinostat (p-value = 0.046), whereas high-risk patients were more sensitive to Dasatinib (p-value = 0.00021, Fig. 6A).

### Functional annotation of the DEGs between two risk groups in the TCGA set

A total of 624 DEGs (FDR < 0.05 and |log_2_FC| > 0.5) were identified between the two risk subgroups of the TCGA set. These genes were significantly enriched in 25 GO biological processes and 10 KEGG signaling pathways, such as inflammatory response, positive regulation of interleukin-6 production, Calcium signaling pathway, MAPK signaling pathway, and IL-17 signaling pathway. Top 10 BP terms and KEGG signaling pathways are showcased in Fig. 6B.

## Discussion

LncRNAs play critical regulatory roles in progression, metastasis, and prognosis of CC [27]. As two well-studied forms of methylation modifications, m^6^A and m^5^C modifications that contribute to cancer occurrence and development are gaining increasing attention [28]. Our study underlined the regulatory mechanisms of m^6^A and m^5^C modifications-related lncRNAs in CC. We identified 62 m^6^A- and m^5^C-related lncRNAs in CC samples, and constructed a co-expression network with these lncRNAs, 23 m^6^A-related genes and 15 m^5^C-related genes. Based on nine survival-related lncRNAs we obtained two molecular subtypes with distinctive TME characteristics. Noticeably, we constructed a prognostic model based on six m^6^A- and m^5^C-related signature lncRNAs, which separated patients into high-risk and low-risk groups with significantly different OS time. Robustness of the prognostic model was successfully validated in an independent cohort. Low-risk patients showed higher sensitivity to AZD8055, Rapamycin and Vorinostat, whereas high-risk patients showed higher sensitivity to Dasatinib. Furthermore, a nomogram based on risk score and prognostic clinical factors was built and showed robust and accuracy performance in predicting prognosis in CC patients. These results suggest that the prognostic model based on six m^6^A- and m^5^C-related lncRNA is a promising tool for prognosis stratification of CC patients and provide new hints to direct individualized therapies.

Previous studies suggest that m^6^A RNA methylation regulators play critical roles.

in the malignant progression of CC, and have prognostic implications [29, 30]. Moreover, m^6^A-related lncRNAs may be promising prognostic biomarkers for CC [14]. The m^6^A- and m^5^C-related lncRNAs signature obtained by our study contained lncRNA ASB16-AS1, DANCR, FBXL19-AS1, LINC01089, ST7-AS1, and ZNF213- AS1. LncRNA ASB16-AS1 is a promising pan-cancer prognostic biomarker, with an association with immune infiltration [31]. There is evidence that lncRNA ASB16-AS1 expression is up-regulated in CC, strengthening cell proliferation, and migration *via* Wnt/β-catenin signal pathway [32]. LncRNA DANCR has prognostic significance in human cancers, and correlates with worse prognosis [33]. Substantial evidences have supported an oncogenic role of lncRNA DANCR in CC, which promotes CC proliferation, metastasis and progression [34, 35]. Emerging studies show that lncRNA FBXL19-AS1 promotes CC proliferation and metastasis [36, 37]. Besides, prognostic potential of lncRNA FBXL19-AS1 has been suggested for CC [38]. LncRNA LINC01089 exerts suppressive effects on the development of CC [39]. LncRNA ST7-AS1 overexpression exhibits positive correlations with shorter OS time, a higher frequency of lymph node metastasis and deeper cervical invasion [40]. Our study consistently found involvement of the 5 m^6^A- and m^5^C-related lncRNAs in the progression of CC. Elevated expression of lncRNA ZNF213-AS1 plays a role in differentiation and proliferation of acute myeloid leukemia and low-grade gliomas, with an insignificant association with poor prognostic outcome [41]. However, biological roles of lncRNA ZNF213-AS1 in CC remain undefined. Our study indicates that these m^6^A- and m^5^C-related lncRNAs may serve as prognostic biomarkers for CC.

Our study revealed two molecular subtypes based on express levels of prognostic lncRNAs. The majority of subtype 1 samples had low-risk scores and longer OS time, whereas the majority of subtype 2 samples had high-risk scores and shorter OS time. TME has been recognized as a principal component of CC tumorigenesis and development, and influences prognosis and treatment of patients [42, 43]. Plasmacytoid dendritic cells play an immunosuppressive role in TME and promote tumor growth [44]. Our study unveiled distinctive TME characteristics of the two subtypes. Subtype 1 had significantly higher levels of activated B cells, CD56 + bright natural killer cells, plasmacytoid dendritic cells, and lowers levels of myeloid derived suppressor cells. It implies that stronger anti-tumor immune function is an important contributor to better prognosis of subtype 1 compared to subtype 2. These findings indicate that m6A- and m5C-related lncRNAs could serve as effective prognostic biomarkers and predictors for clinical outcomes and immunotherapeutic responses in CC patients. Additionally, our study found that the low-risk patients were more sensitive to AZD8055, Rapamycin and Vorinostat, whereas high-risk patients were more sensitive to Dasatinib. It offers valuable data to facilitate individualized clinical treatments for CC patients. Our study also unraveled that various inflammation-related biological processes and pathways, such as inflammatory response, positive regulation of interleukin-6 production, and IL-17 signaling pathway, as well as Calcium signaling pathway and MAPK signaling pathway might participate in the regulatory mechanisms of m^6^A and m^5^C modifications-related lncRNAs in CC in concordance with previous findings [45–47].

This study has several limitations. First, since the data were analyzed from the TCGA and GEO databases, there is a lack of validation through experiments. Second, the prognostic value of the RiskScore model requires validation with additional external datasets before it can be applied clinically. Finally, the potential molecular mechanisms of these m^6^A/m5C-related lncRNAs in cervical cancer remain unclear, and we plan to conduct in vitro or in vivo experiments in future research to validate our findings.

## Conclusion

Taken together, our study comprehensively analyzed interactions of m^6^A and m^5^C modifications with lncRNAs in CC samples, and established a prognostic model based on a signature of six m^6^A- and m^5^C-related lncRNAs, which could be used to predict outcome of patients. These signature lncRNAs had close associations with TIICs and cancer prognosis, highlighting great promises as prognostic biomarkers and therapeutic targets for CC. Our study expands the knowledge concerning involvement of m^6^A- and m^5^C-related lncRNAs in CC pathogenesis and has clinical implications for risk stratification and personalized therapeutics of patients.

## Electronic supplementary material

Below is the link to the electronic supplementary material.

Supplementary Material 1

Supplementary Material 2

## Data Availability

The data that support the findings of this study are available from the corresponding author upon reasonable request.

## Abbreviations

(m6A): N6 methyladenosine
(m5C): 5 methylcytosine
(lncRNAs): long non-coding RNAs
(CC): cervical cancer
(TIICs): tumor infiltrating immune cells
(OS): overall survival
(CESC): cervical squamous cell carcinoma
(HPV): Human papilloma virus
(mRNAs): messenger RNA
(ncRNAs): non-coding RNAs
(TCGA): The Cancer Genome Atlas
(TIICs): tumor infiltrating immune cells
(GEO): gene expression omnibus
(KM): Kaplan- Meier
(ROC): receiver operating characteristic
(GDSC): Genomics of Drug Sensitivity in Cancer
(DEGs): Differentially expressed genes
(GO): Gene ontology
(KEGG): Kyoto Encyclopedia of Genes and Genomes
(DAVID): Database for Annotation, Visualization and Integrated Discovery
