# Supplementary material for: Integrated analysis of N6-methyladenosine- and 5-methylcytosine-related long non-coding RNAs for predicting prognosis in cervical cancer
Source: Hereditas. 2024 Sep 16;161:34. doi: 10.1186/s41065-024-00336-w (PMC11403863; doi:10.1186/s41065-024-00336-w)
Supplement: Supplementary file 1 — Supplementary Material 1 [file 41065_2024_336_MOESM1_ESM.docx]

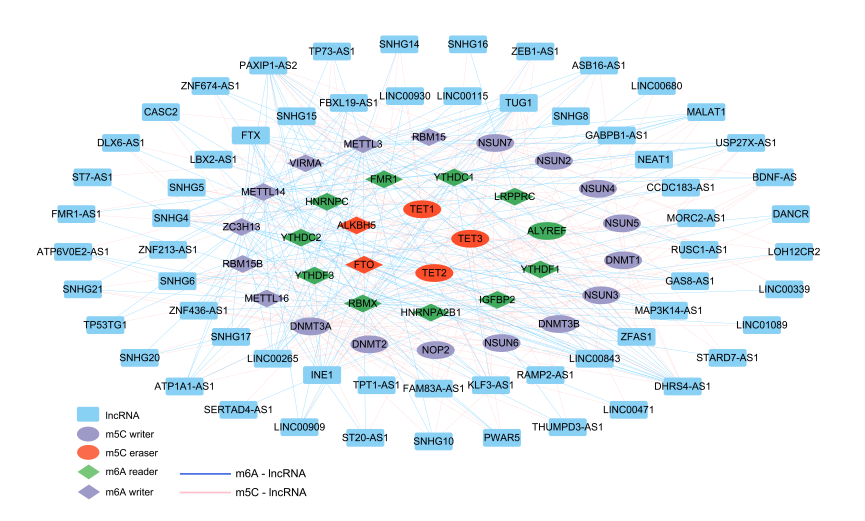


**Figure S1** A co-expression network of m^6^A regulators, m^5^C regulators and lncRNAs.

The network is composed of 23 m^6^A regulators, 15 m^5^C regulators and 343 lncRNAs.
