# Supplementary material for: Integrated analysis of N6-methyladenosine- and 5-methylcytosine-related long non-coding RNAs for predicting prognosis in cervical cancer
Source: Hereditas. 2024 Sep 16;161:34. doi: 10.1186/s41065-024-00336-w (PMC11403863; doi:10.1186/s41065-024-00336-w)
Supplement: Supplementary file 2 — Supplementary Material 2 [file 41065_2024_336_MOESM2_ESM.docx]

Supplemental Table 1 m^6^A regulators and m^5^C regulators

| m^6^A regulators | Type | m^5^C regulators | Type |
| --- | --- | --- | --- |
| METTL3 | writers | NOP2 | writer |
| METTL14 | writers | NSUN2 | writer |
| METTL16 | writers | NSUN3 | writer |
| WTAP | writers | NSUN4 | writer |
| VIRMA | writers | NSUN5 | writer |
| ZC3H13 | writers | NSUN6 | writer |
| RBM15 | writers | NSUN7 | writer |
| RBM15B | writers | DNMT1 | writer |
| YTHDC1 | readers | DNMT2 | writer |
| YTHDC2 | readers | DNMT3A | writer |
| YTHDF1 | readers | DNMT3B | writer |
| YTHDF2 | readers | TET1 | eraser |
| YTHDF3 | readers | TET2 | eraser |
| HNRNPC | readers | TET3 | eraser |
| FMR1 | readers | ALYREF | reader |
| LRPPRC | readers |  |  |
| HNRNPA2B1 | readers |  |  |
| IGFBP1 | readers |  |  |
| IGFBP2 | readers |  |  |
| IGFBP3 | readers |  |  |
| RBMX | readers |  |  |
| FTO | erasers |  |  |
| ALKBH5 | erasers |  |  |
